# Characterization of *Bacillus thuringiensis* isolates by their insecticidal activity and their production of Cry and Vip3 proteins

**DOI:** 10.1371/journal.pone.0206813

**Published:** 2018-11-01

**Authors:** Burcu Şahin, Joaquín Gomis-Cebolla, Hatice Güneş, Juan Ferré

**Affiliations:** 1 Department of Biology, Faculty of Science, Muğla Sıtkı Koçman University, Muğla, Turkey; 2 ERI de Biotecnología y Biomedicina (BIOTECMED), Department of Genetics, Universitat de València, Burjassot, Spain; Nigde Omer Halisdemir University, TURKEY

## Abstract

*Bacillus thuringiensis* (*Bt*) constitutes the active ingredient of many successful bioinsecticides used in agriculture. In the present study, the genetic diversity and toxicity of *Bt* isolates was investigated by characterization of native isolates originating from soil, fig leaves and fruits from a Turkish collection. Among a total of 80 *Bt* isolates, 18 of them were found carrying a *vip3* gene (in 23% of total), which were further selected. Insecticidal activity of spore/crystal mixtures and their supernatants showed that some of the *Bt* isolates had significantly more toxicity against some lepidopteran species than the HD1 reference strain. Five isolates were analyzed by LC-MS/MS to determine the Cry protein composition of their crystals. The results identified the Cry1Ac protein and a Cry2A-type protein in all isolates, Cry1Ea in 3 of them and Cry1Aa in one. The sequence analysis of the new *vip3* genes showed that they had a high similarity to either *vip3Aa*, *vip3Af* or *vip3Ag* (94–100%). The *vip3Aa* gene of the 6A *Bt* isolate was cloned and sequenced. The protein was named Vip3Aa65 by the *Bacillus thuringiensis* Nomenclature Committee. The expressed and purified Vip3Aa65 protein was tested against five lepidopteran species and its toxicity compared to that of a reference protein (Vip3Aa16). Both proteins had similar toxicity against *Grapholita molesta* and *Helicoverpa armigera*, whereas Vip3Aa65 was less active than Vip3Aa16 against three species from the *Spodoptera* genus. A tetrameric structure of the Vip3Aa65 protein was detected by gel filtration chromatography. The study revealed some isolates with high insecticidal activity which can be considered promising candidates to be used in pest control.

## Introduction

*Bacillus thuringiensis* (*Bt*) is a gram positive entomopathogenic bacterium widely used in biological control against various economically important pests. *Bt* products are used in pest management because they are highly specific to the target organism and harmless to non-target organisms and the environment [1, 2]. Because of their effectiveness to control insect pests, they represent the 90% of the bioinsecticide market [3]. The insecticial proteins produced by *Bt* have been classified into those that accumulate in the parasporal inclusions at stationary growth phase, such as Cry and Cyt proteins (σ-endotoxins), and the ones that are secreted during the vegetative growth (Vip and Sip) [2]. Most *Bt* commercial spray products contain either Cry proteins, or a combination of Cry and Cyt proteins in the case of mosquitocidal products. Transgenic plants protected from insects also express *Bt* proteins (the so called *Bt* crops), mainly Cry and Vip3 proteins. Cry proteins are mainly toxic to Lepidoptera, Coleoptera and Diptera. Vip3 proteins, whose sequence homology is completely unrelated to that of Cry proteins, have toxicity to a broad variety of lepidopteran pests [4, 5].

Since genes coding for the insecticidal proteins are usually carried on plasmids, a high diversity in toxin genes and their combination are found in *Bt* isolates due to plasmid transfer among them followed by mutation [6]. Therefore, the insecticidal potency of isolates in *Bt* collections, obtained from different geographies all over the world, has been examined, with the result of finding out very active strains that may make up the active ingredient of new commercial products [7, 8, 9]. The study of the gene content in isolates of such collections has led to the discovery of novel genes and variations in the already existing ones [10]. These studies are important to broaden the list of target pests of *Bt* products and also to find out new toxins to help overcome the resistance developed by insects against currently applied toxins.

The aim of the current study was to characterize native *Bt* isolates from a Turkish collection and to select those that would combine a good insecticidal potential in their parasporal crystal along with the production of Vip3 proteins in the supernatant. One isolate with high expresion of the Vip3 protein and high toxicity in the supernatant, was chosen to clone a new *vip3* gene, which was further characterized. The new Vip3 protein was expressed, purified and tested against the beet armyworm (*Spodoptera exigua*), cotton leafworm (*Spodoptera littoralis*), fall armyworm (*Spodoptera frugiperda*), cotton bollwarm (*Helicoverpa armigera*), and oriental fruit moth (*Grapholita molesta*).

## Materials and methods

### DNA isolation and detection of the *vip3*-type genes

Total genomic DNA was isolated from a single colony of the *Bt* isolates as described by Ferrandis et al. [8]. The DNA was quantified in a Nanodrop 2000 (Thermo Scientific) and the integrity was evaluated by agarose gel electrophoresis (1% agarose). The presence of *vip3* genes was performed by amplifying the N-terminal part of the *vip3* gene with screening primers (Table 1). The PCR reaction contained, in a final volume of 25 μl, 100 ng of DNA template, 2.5 μl 10× reaction buffer, 0.2 mM of each dNTP, 1 U *Taq* DNA polymerase (BIOTOOLS B&M Labs), 0.2 μM of forward and reverse primers. PCR amplifications were performed in an Eppendorf Mastercycler thermal cycler as follows: 4 min of denaturation at 94°C, followed by 35 cycles of amplification (40 s denaturation at 94°C, 1 min of annealing at 50°C, 2 min of extension at 72°C) and a final extension step of 7 min at 72°C.

**Table 1:** Primer pairs used for identification and sequencing of *vip3* gene in the *Bt* isolates.

	Primers	Sequence^*a*^ (5’-3’)	Nucleotide position in the reference sequence	Amplicon size (bp)	References
**Screening primers**	*vip3-sc(f)*	TGCCACTGGTATCAARGA	-	-	[10]
	*vip3-scII(r)*	CCATTAATYGGAKTCAAAAATGTTTCACTGAT	78–1472	1395	[11]
**Typing primers**	*vip3A-F1*	ASTTTAAGATATGAGGYAACAGC	-	-	In this work
	*vip3Aa-R1*	CATCGTAAAAATGTACAATAGGA	1297–2356	1060	In this work
	*vip3Af-R1*	TCAAATGATATATGACCACCA	1297–2348	1052	In this work
	*vip3Ag-R1*	ATGTAAAACGAGAAAGCTCTACA	1297–2323	1027	In this work

^a^ Universal code for degenerate bases: R = A, G; Y = C, T; K = G,T; S = G, C.

### Preparation of spore/crystal mixtures and supernatants for bioassays

*Bt* isolates were grown on CCY medium agar [12] at 29°C for 48 hours. To obtain the spore/crystal mixtures, one loop of each *Bt* colony was transferred into 0.5 ml sterile distilled water. The sample was heated at 70°C for 30 min in order to synchronize and eliminate vegetative forms in the culture. Then, the suspension was transferred to 10 ml of CCY liquid medium and incubated at 30°C for 48–72 hours with 250 rpm shaking. Spore and crystal formation was checked by phase-contrast microscope, and then the suspension was centrifuged at 9700 ×*g* for 10 min. The pellet was resuspended in 5 ml ultrapure water and centrifuged again. This process was repeated twice. Then, the pellet was resuspended in 5 ml ultrapure water and kept at −20°C.

To obtain the supernatants for the bioassays, a colony of each *Bt* isolate was inoculated into 10 ml of LB medium. After incubation at 30°C for 24–36 h with shaking (250 rpm), the suspension was centrifuged at 9700 ×*g* for 10 min. The supernatant fraction was kept at −20°C.

### Toxicity assays with spore/crystal mixtures and supernatants

Surface contamination assays of spore/crystal mixtures and supernatants were performed against *S*. *littoralis*, *S*. *exigua* and *Ostrinia nubilalis* neonate larvae in a bioclimatic chamber at 25°C, 60 ± 10% RH, 16:8 light/dark photoperiod. For spore/crystal mixtures a preliminary assay was performed at a single dose adjusting its concentration to 0.6 OD at 600 nm. The mixture suspension was applied (50 μl) onto artificial diet based on corn flour and wheat germ, and containing yeast, ascorbic acid and nipagin [13,14,15] (2 cm^2^ multiwell plates) and allowed to dry in a flow hood. Sterile ultrapure water and *B*. *thuringiensis* ssp. *kurstaki* strain HD1 were used as negative and positive controls, respectively. The most active isolates were further tested in dose response assays. First, the OD units of spore/crystal mixtures were converted to concentration units (mg crystal protein per ml). For this, the crystal proteins in the pellet of the mixture were dissolved in carbonate buffer (pH 10.5) by incubation for 2 hours at 37°C with shaking (100 rpm). After centrifugation at 16,000 ×*g* for 15 min at 4°C, the concentration of crystal protein in the supernatant was determined by the Bradford assay [16]. The assays were performed in 3 replicates with 5 serial dilutions (3-fold dilutions starting with an OD of 0.6 at 600 nm), using 16 neonate larvae for each concentration. Mortality was recorded after 7 days and statistical analyses were performed with the Polo-PC Programme (LeOra Software, Berkeley, CA). LC_50_ values were considered significantly different when fiducial limits did not overlap.

Bioassays with the supernatant fraction were carried out by applying 50 μl of the straight supernatant onto the diet. LB medium was used as negative control. Sixteen neonate larvae were used for each treatment. Mortality was recorded after 10 days.

### β-exotoxin analysis in the supernatant of *Bt* isolates

Type I β-exotoxin detection in the supernatant of the fermentation broth was performed by LC-MS/MS analysis at the proteomics facility of SCSIE (Servei Central de Suport a la Investigació Experimental) at the University of Valencia. A single colony was inoculated into 10 ml of CCY medium and incubated for 48 h at 28°C, 250 rpm. Then, the culture was centrifuged at 9000 ×*g* for 10 min and the supernatant was filtrated through a 0.45 μm cellulose acetate filter. The *B*. *thuringiensis* var. *thuringiensis* strain MA10.30 (lab stock) and the *B*. *thuringiensis* var. *kurstaki* strain HD1 were used as positive and negative control of β-exotoxin, respectively. β-exotoxin was detected by looking for the transitions 702.1–136.2 and 702.1–412.0 in the chromatogram peaks [17].

### Detection and sequencing of *vip3*-type genes

The PCR products generated by the screening primers [10,11], covering the N-terminal part of gene, were purified and sequenced. The sequence results were analyzed with DNAstar v5 and NCBI Blast tools. To cover most of the full length of the gene we designed typing primer sets, which amplified the C-terminal part of the *vip3* gene. The PCR was performed as described above with the screening primers, except for the amplification cycles which consisted of 1 min denaturation at 94°C, 1 min of annealing at 52°C, and 2 min of extension at 72°C. The PCR products were purified and sequenced and the results combined with those obtained with the screening primers using the EMBOSS programme and analyzed with BLAST tools.

### Analysis of expression of the *vip3*-type genes from *Bt* isolates

Vip3A protein expression was determined with a dot blot assay. A single *Bt* colony was inoculated to 4 ml LB medium and incubated at 29°C for 36–48 h at 250 rpm. The OD of all the cultures was adjusted to a value of 1.05 by adding LB medium and then the culture was centrifuged at 9700 ×*g* for 10 min at 4°C. The supernatant was filtered through 0.45 μm and then 0.22 μm cellulose acetate filters. The filtered supernatant (15 μl) was applied onto the nitrocellulose membrane. The membrane was blocked with 5% skimmed milk in PBST (Phosphate Buffered Saline 1×, 0.1% Tween 20) for 1 h at room temperature. The membrane was washed 3 times with PBST for 5 minutes and incubated with anti-Vip3Aa antibody at 1:5,000 dilution (v/v) in PBST containing 2% skimmed milk for 2 h. After the incubation with the anti-Vip3Aa antibody, the membrane was washed three times with PBST for 5 min and further incubated with anti-rabbit IgG antibody-alkaline phosphatase (Sigma-Aldrich) at 1:10,000 dilution (v/v) as above. The membrane was washed three times with PBST for 5 minutes and developed with Western Blotting Detection System (GE Healthcare, Uppsala, Sweden) with Image Quant LAS 400. The Vip3Aa antibody was obtained by injecting rabbits with a solution of Vip3Aa16; this antibody cross-reacted with other Vip3A proteins [18].

### Protein preparation for LC-MS/MS analysis

*Bt* isolates were grown on CCY agar plates at 29°C for 48 h. A single colony was inoculated in 5 ml of CCY medium and incubated until sporulation at 29°C at 180 rpm. The culture growth was synchronized by heating at 70°C for 20 min before inoculation of 0.1 ml into 100 ml of CCY medium (ratio 1:1000) and this was further incubated at 29°C for 48 h at 180 rpm. The spore/crystal mixture was collected by centrifugation at 6000 ×*g* for 12 min at 4°C. The pellet was washed two times with solution A (1 M NaCl, 10 mM EDTA, 10 mM PMSF, 1% Triton X 100) and then centrifuged at 17,000 ×*g* at 4 ^o^C for 12 min. The pellet was washed twice in solution B (10 mM KCl, 10 mM PMSF) and centrifuged at 24,000 ×*g* at 4°C for 15 min. The crystal proteins were solubilized in 50 mM carbonate buffer, pH 10.5, 10 mM DTT, for 2 h at room temperature with gentle shaking. After that, solubilized proteins were centrifuged at 24,000 ×*g* for 15 min at 4°C. The supernatant was filtered through 0.45μm cellulose acetate filter and kept at −20°C. Protein concentration of the samples was determined by the Bradford assay [16].

### Determination of the cry protein content in *Bt* isolates based on their unique peptides

The detection of the Cry protein content in the *Bt* isolates was done by LC-MS/MS at the proteomics facility of the SCSIE (Servei Central de Suport a la Investigació Experimental) at the University of Valencia. To further prepare the samples for LC-MS/MS analysis, 20 μg of the samples were dried in a rotatory evaporator. Then, the samples were solubilized in a final volume of 20 μl of 50 mM ammonium bicarbonate containing 2 mM DTT, for 20 minutes at 60°C. The thiol groups were alkylated by 5.5 mM of iodoacetamide (IAM), in a final volume of 30 μl, for 30 min at room temperature in the dark. The excess of IAM was quenched with 10 mM DTT (60 μl final volume) at 37°C for 1 h. The samples were digested overnight with 500 ng of trypsin and the digestion was stopped with 6 μl of 10% trifluoroacetic acid (TFA) in water. The LC/MSMS analysis was performed in an MSMS nanoESI qQTOF 5600 TripleTOF (Ab SCIEX) as described earlier [19]. The protein identification of the solubilized crystal proteins was carried out by the Paragon algorithm [20] via the Protein Pilot v 5.0 (ABSciex). Protein Pilot v 5.0 default parameters (trypsin specificity, cys-alkylation, taxonomy restricted to human, and the search effort set to through) were used to search in the SwissProt protein database. False positive level <1% was estimated by target-decoy search.

The LC-MS/MS analysis is a common technique for protein identification in simple protein mixture, such as *Bt* crystals that contain few Cry proteins in the same crystal like Cry1 and Cry2. Due the high similarity of the Cry1 and Cry2 proteins, the LC-MS/MS analysis can provide false positives in the identification of proteins within the Cry1 or Cry2 protein families, but not between Cry1 or Cry2 protein families. The unique peptides of the identified Cry proteins were determined as follows: (1) in the protein summary, the crystal proteins showing an unused score higher than 2 (protein identified with a confidence higher than >99%) were selected. (2) In the peptide summary, for those proteins with and unused score higher than 2, we selected those peptides with the following criteria: number of amino acids range 6 to 20, contribution value higher than 2 and without modifications. To avoid the redundancy in the peptides used for the protein identification, we compared the filtered peptides and selected those that only were found once. Regarding the peptides that were repeated, we selected only one member of each group. (3) The peptide list of each protein was analyzed using Blastp, NCBI Blast tools, against the reference sequence of the Cry proteins identified in the first point. Those peptides that were only found once in one protein were considered as “unique peptides” (S1 Table), while the peptides that were found in two or more proteins were considered “shared peptides”.

We define as a “positive identification” all those Cry proteins that, at least, the protein identification was supported by two unique peptides.

### Cloning and sequencing of the *vip3*-type genes in *Escherichia coli*

The complete open reading frame (ORF) of the *vip3-*type gene (2.4 kb) from the 6A isolate was amplified with forward Vip3Aa45expF (5’-*CGC*GGATCCATCGAAGGTCGTATGAACAAGAATAATACTAAAT-3’) and reverse Vip3Aa45expR (5’-*AAGGAAAAAA*GCGGCCGCTTACTTAATAGAGACATCGTAA-3’) primers. *Bam*HI and *Not*I restriction sites and extra nucleotides flanking *BamH*I and *Not*I were added to the 5’-ends of forward and reverse primers, respectively (single underlined and italics in the sequence, respectively). Also, a Factor Xa protease cleavage site (double underlined) was added to the forward primer. In a final volume of 25 μl, PCR mixtures included 100 ng of the DNA template, 0.5 U of KAPA HiFi DNA polymerase, 5 μl of 5× reaction buffer, 10 mM KAPA dNTP mix, and 0.3 μM of the gene specific primers. Amplifications were carried out in an Eppendorf Mastercycler thermal cycler. The PCR was performed as follows: 4 min denaturation at 94°C followed by 35 cycles of amplification with 1 min denaturation at 94°C, 1 min of annealing at 55°C, and 2 min of extension at 72°C. An extra extension step of 7 min at 72°C was added. The *vip3-*type gene was cloned into the expression vector pET-30a(+). For this, purified insert and vector DNA was digested with *Bam*HI and *Not*I enzymes to create sticky ends. Ligation was performed with 3:1 (insert: vector) ratio by using Thermo T4DNA Ligase (5U/μl). The ligation product was then transformed into competent *E*. *coli* DH5α cells [21]. Colony PCR was performed with selected colonies. Then, plasmid DNA of the positive colonies was isolated (Nucleospin Plasmid Kit) and digested with *Bam*HI and *Not*I to confirm the presence of the vector and insert DNA. Then, plasmid DNA of the positive colonies was sequenced using T7 terminator, T7 universal, *vip3* internal 1 (5’-GATGTAATGAAACAAAATTATG-3’) and *vip3* internal 2 (5’-CTAAAACAAATTATCAAGTCG-3’) primers in order to check if the *vip3Aa* gene is in frame in the pET-30a(+) vector.

### Protein expression and purification of Vip3Aa proteins cloned in *E*. *coli*

The vector containing the *vip3Aa65* gene was transformed to BL21(DE3) competent cells. The recombinant *E*. *coli* BL21(DE3) cells were spread into LB-kanamycin (50 μg/ml) agar plate and incubated at 37°C overnight. One single colony was inoculated in a preculture containing 20 ml of LB-kanamycin medium (50 μg/ml), and grown overnight at 37°C, 200 rpm. The preculture (5 ml) was transferred to 500 ml LB medium containing kanamycin (50 μg/ml) and incubated at 37°C, 180 rpm. When the OD_600_ reached 0.6–0.9, 1 mM IPTG (isopropyl-b-D-thiogalactopyranoside) was added for induction. The culture was grown overnight at 37°C, 180 rpm. Cells were centrifuged at 8,800 ×*g* for 30 min at 4°C. The pellet was resuspended in lysis buffer (20 mM phosphate buffer, 0.5 M NaCl, pH 7.4) containing 100 μM PMSF, 3 mg/ml lysozyme, and 10 μg/ml DNAse, and incubated with shaking for 30 min at 37°C. The sample was sonicated twice for 60 s, with a 10 s pause in between. The supernatant was collected following centrifugation at 27,000 ×*g* for 30 min and filtered through a 0.22 μm cellulose acetate filter.

The *vip3Aa16* gene was kindly provided by the Laboratory of Biopesticides (Centre de Biotechnologie de Sfax, Tunisia). The gene had been cloned into pET vector with a His-tag and then subcloned into *E*. *coli* BL21 [22]. The Vip3Aa16 protein was expressed as reported earlier with minor modifications [23]. After induction with IPTG, the cells were lysed and the supernatant containing the Vip3Aa16 protein was recovered as described above for the Vip3Aa65 protein.

Vip3Aa proteins were purified by HisTrap FF crude Lysate columns (GE Healthcare) precharged with NiSO_4_. The column was equilibrated with phosphate-NaCl buffer (50 mM phosphate, pH 7.4, 300 mM NaCl) containing 10 mM imidazol. Then the lysate was loaded onto the column and washed with phosphate-NaCl buffer with 45 mM of imidazole. The Vip3Aa protein was eluted with phosphate-NaCl buffer containing 150 mM of imidazole. Fractions (1 ml) were collected in tubes containing 50 μl of 0.1 M EDTA. The purity and amount of Vip3Aa in the fractions was determined by SDS-PAGE and the most concentrated fractions were combined. In the case of Vip3Aa65, the N-terminal tags were removed with Factor Xa protease after dialysis against 20 mM Tris-HCl buffer, pH 8, 100 mM NaCl and 2 mM CaCl_2_ for 2 h. At this point, both Vip3Aa65 and Vip3Aa16 were dialyzed overnight against 20 mM Tris buffer, pH 8.6, 150 mM NaCl and 5 mM EDTA. Protein concentration was determined by Bradford and the purified proteins were kept at −20°C until used.

### Gel filtration chromatography of the Vip3Aa65 protein

The apparent molecular weight in native conditions of the full length protein (protoxin) and the trypsin-processed protein (activated toxin) was determined by gel filtration chromatography. To obtain the trypsin-activated toxin, the Vip3Aa65 protein was incubated with trypsin at a 24:100 ratio (trypsin: protoxin) for 6 h at 37°C. Chromatography was performed in an ÄKTA explorer 100 chromatography system (GE Healthcare) using a Superdex 200 10/300 GL column equilibrated with 50 mM Tris-HCl, 300 mM NaCl, pH 9, at a flow rate of 0.5 ml/min with detection at 280 nm. The sample injection volume was 0.3 ml and the Vip3Aa concentration 1 mg/ml (for both samples). The column had been previously calibrated with the gel filtration calibration kit HMW (high molecular weight, GE Healthcare). Elution fractions were collected and aliquots run on SDS-PAGE.

### Dose-response assays with purified Vip3Aa proteins

Neonate larvae of *S*. *exigua*, *S*. *littoralis*, *S*. *frugiperda*, *H*. *armigera a*nd *G*. *molesta* were used to test the insecticidal activity of purified Vip3Aa65 and Vip3Aa16 proteins. Dose response assays of seven different serial dilutions (concentrations were chosen as to include the LC_50_ for each insect species) were conducted following the procedure described for spore/crystal mixtures assays. The buffer in which the proteins were stored was used as a negative control.

## Results

### Selection of *Bt* isolates carrying *vip3* genes

**Fig 1:**
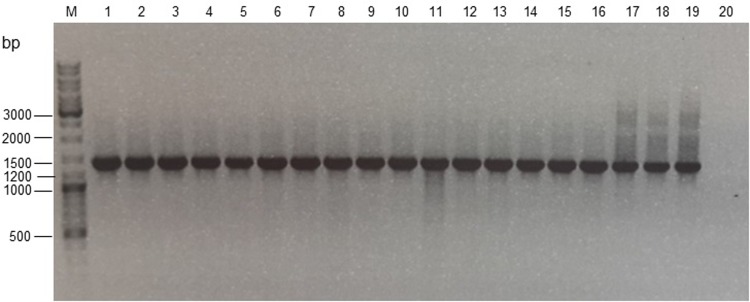
Agarose gel electrophoresis of PCR products with *vip3-sc(f)* and *vip3-scII(r)* primers of *vip3* positive *Bt* isolates. M: DNA Ladder, 1: Positive control (HD1), 2: 6A, 3: 42MY, 4: 13MY, 5: 45MY, 6: Bt-KE63-64, 7: Bt-KH3; 8: Bt-KH58, 9: Bt-BY7, 10: 85PPb, 11: 125Q, 12: 43MY, 13: 44MY, 14: 51MY; 15: 70MY, 16: 19Q2, 17: 19Q3, 18: 60Q1, 19: 60Q2, 20: Negative control.

Eighty isolates in a *Bt* collection were screened for the presence of *vip3* genes by PCR using screening primers (Table 1). Among them, 18 *Bt* isolates were found positive to carry a *vip3* gene and were selected for further characterization. Fig 1 shows their PCR products (1395 bp) after agarose gel electrophoresis.

### Insecticidal characterization of *vip3*-selected isolates

The spore/crystal mixtures and supernatants of the 18 *Bt* selected *vip3*-positive isolates were tested for their insecticidal activity against *S*. *littoralis*, *S*. *exigua* and *O*. *nubilalis* neonate larvae and compared with those of the HD1 strain, used as a reference since it is present in many commercial bioinsecticides.

For the spore/crystal fraction, a preliminary screening at a single dose allowed us to select those isolates with apparently higher insecticidal activity than the HD1 strain, which were further tested in dose-response assays (Table 2). In the case of *S*. *exigua*, only one Bt isolate, Bt-KH58, was significantly more toxic (LC_50_ = 15 ng/cm^2^, FL = 9–24) than the HD1 strain (LC_50_ = 48 ng/cm^2^, FL = 30–74). For *S*. *littoralis*, four isolates showed higher activity than HD1 (6 to 12-fold). On the other hand, none of the isolates was found significantly more toxic than HD1 against *O*. *nubilalis*.

**Table 2:** Dose-response assays of spore/crystal mixtures of selected *Bt* isolates^a^.

	*S*. *exigua*		*S*. *littoralis*		*O*. *nubilalis*	
Isolate	Slope±SE	LC_50_ (ng/cm^2^) FL95% (min.-max.)	Slope±SE	LC_50_ (ng/cm^2^) FL95% (min.-max.)	Slope±SE	LC_50_ (ng/cm^2^) FL95% (min.-max.)
**HD1**	1.05±0.13	48 (30–74)	1.07±0.12	15.6 (8.7–28.1)	1.45±0.19	6.7 (3.2–11.5)
**6A**	1.94±0.24	113 (83–154)	1.61±0.18	19 (12–31)	1.82±0.22	3.5 (2.0–5.7)
**13MY**		-	1.54±0.20	1.31 (0.59–2.18)		-
**19Q2**	1.76±0.23	270 (175–444)		-		-
**19Q3**		-		-		-
**42MY**	2.39±0.26	51 (26–92)	1.62±0.21	2.8 (1.4–4.4)	1.39±0.18	4.5 (2.0–7.8)
**43MY**		-		-	1.44±0.17	5.8 (2.8–10.7)
**44MY**		-		-	1.81±0.22	2.5 (1.5–4.0)
**45MY**	2.11±0.24	33 (25–42)	1.75±0.19	1.57 (0.94–2.50)	1.26±0.13	3.3 (2.1–5.3)
**51MY**	1.37±0.16	23 (12–42)	1.27±0.19	2.21 (0.55–4.44)	1.71±0.21	4.5 (2.1–9.1))
**60Q1**		-		-	1.15±0.15	28 (14–52)
**60Q2**		-		-	1.22±0.15	27 (14–52)
**70MY**		-	1.51±0.16	27 (17–41)	1.67±0.21	5.1 (1.9–11.3)
**85PPb**		-	1.40±0.17	5.1 (2.7–9.2)		-
**125Q**		-		-		-
**Bt-BY7**	1.27±0.15	29 (10–72)	1.32±0.17	11.8 (6.0–19)	1.46±0.17	4.2 (2.8–6.7)
**Bt-KE63-64**		-	1.20±0.17	11.7 (5.9–22)		-
**Bt-KH3**	1.19±0.16	58 (24–116)		-	1.79±0.22	5.5 (3.8–7.6)
**Bt-KH58**	1.20±0.16	15 (9–24)	1.14±0.14	11.0 (6.9–16)	1.19±0.11	8.1 (4.7–13.4)

^*a*^ Only those isolates that showed higher insecticidal activity than the HD1 reference strain were selected for the dose-response assays

The supernatant fraction was tested as both non-treated and autoclaved supernatant. The latter was meant to be a control for insecticidal non-proteinaceus molecules. Table 3 shows the results obtained with those non-treated supernatants that showed toxicity against neonates of the three lepidopteran species tested. No mortality was observed with autoclaved supernatants indicating that the insecticidal activity observed in the non-treated samples was due to a heat-labile molecule, most likely a protein. In addition, direct β-exotoxins analysis of supernatants of these isolates did not reveal the presence of this heat-stable metabolite (data not shown).

**Table 3:** Average percent mortality of neonates to non-treated (NT) and autoclaved (AC) supernatants of *Bt* isolates.

	% Mortality^*a*^					
	*S*. *exigua*		*S*. *littoralis*		*O*. *nubilalis*	
	NT	AC	NT	AC	NT	AC
**HD1**	56	6.3	69	3.1	44	6.3
**6A**	90	0	91	13	19	-
**42MY**	31	-	91	9.4	6.3	-
**45MY**	97	0	91	6.3	0	-
**51MY**	56	-	91	6.3	50	6.3
**Bt-KH3**	31	-	94	6.3	31	0
**Bt-KH58**	13	-	38	-	56	9.4
**LB medium**	3	0	0	13	3.1	0

^*a*^ Assays were performed in 2 replicates for *S*. *exigua* and *S*. *littoralis*, and 4 replicates for *O*. *nubilalis*.

### Characterization of *vip3* genes in selected strains

The PCR products (1395 bp) of *vip3*-positive isolates were sequenced and the results compared to those in databases. Except for two of the isolates whose amplicon could not be sequenced, the rest of the amplicons showed 97–100% identity to either *vip3Aa*, *vip3Af* or *vip3Ag* genes. To obtained a more complete comparative analysis of the new gene sequences with those existing in databases, samples were subjected to PCR amplification of the C-terminal part of the gene with the typing primers. The merged sequences of both the N-terminal part and C-terminal part of the *vip3* genes identified, with more accuracy, the matched genes and their percent identity (Table 4). The results showed identities from 98 to 100% to already existing *vip3Aa*, *vip3Af* and *vip3Ag* genes, except for the 60Q2 isolate which showed 94% identity to the *vip3Ag* gene.

**Table 4:** Percent identity of PCR merged sequences^a^ with matched genes.

Isolate	Identity (%)	Query cover (%)	BLAST hit
**6A**	99	100	*vip3Aa*
**13MY**	100	100	*vip3Aa*
**19Q2**	100	100	*vip3Aa*
**19Q3**^***b***^	-	-	-
**42MY**	100	100	*vip3Aa*
**43MY**	100	100	*vip3Aa*
**44MY**	99	100	*vip3Aa*
**45MY**	99	100	*vip3Aa*
**51MY**	98	99	*vip3Aa*
**60Q1**	99	100	*vip3Af*
**60Q2**	94	99	*vip3Ag*
**70MY**^***b***^	-	-	-
**85PPb**	99	99	*vip3Af*
**125Q**	98	100	*vip3Ag*
**Bt-BY7**	100	100	*vip3Aa*
**Bt-KE63-64**	99	100	*vip3Af*
**Bt-KH3**	99	100	*vip3Aa*
**Bt-KH58**	100	100	*vip3Aa*

^*a*^ Contigs from the PCR sequence of the N-terminal part and the C-terminal part were combined.

^*b*^ No conclusive results from sequencing.

### Vip3A and cry protein expression of selected *Bt* isolates

**Fig 2:**
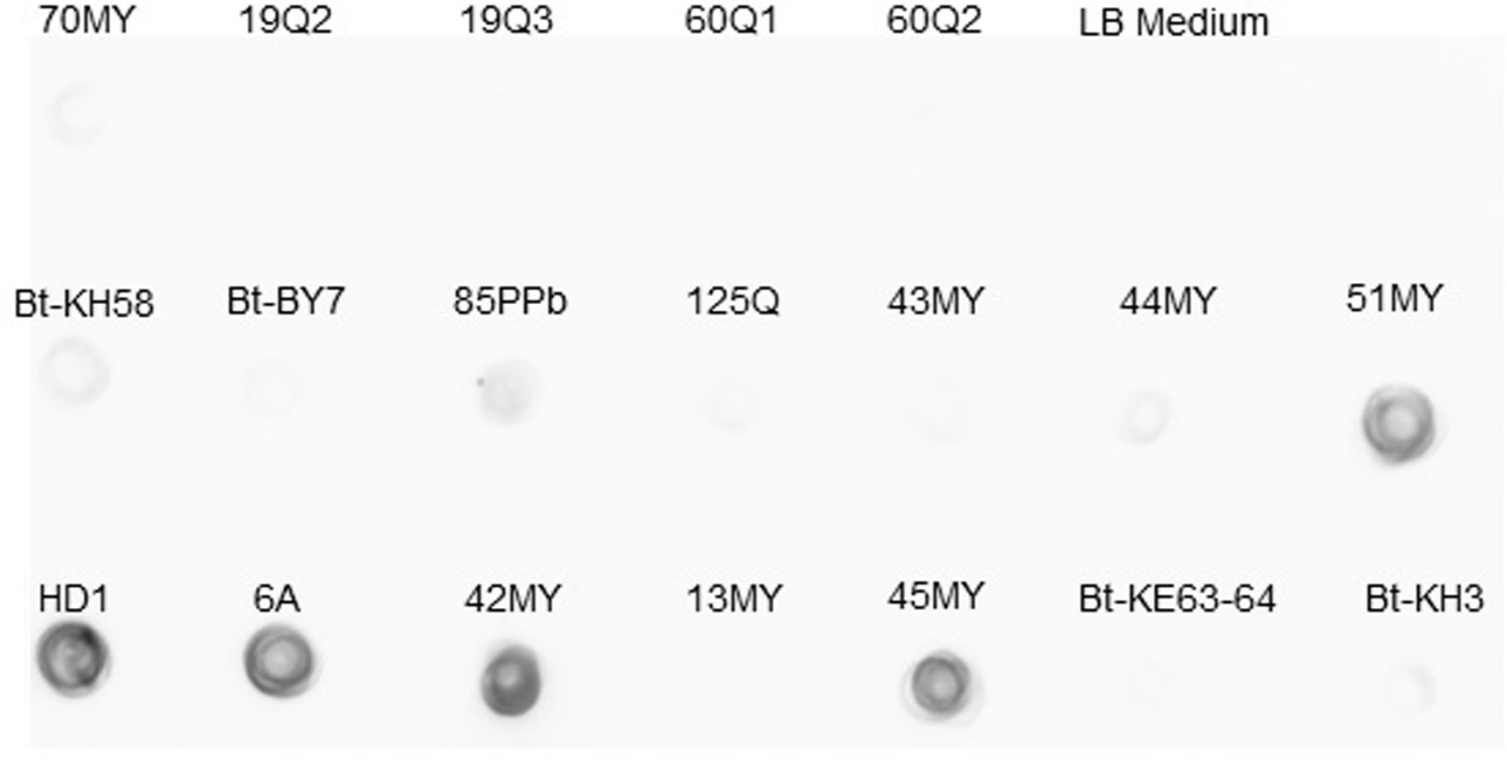
Expression of Vip3A proteins in supernatants of *Bt* isolates. HD1 was used as a positive control and the LB medium as negative control.

Expression of Vip3A proteins was tested in the *vip3*-containing isolates by dot-blot using an anti-Vip3Aa antibody. Seven out of the 18 isolates showed expression of a Vip3A protein (Fig 2). Comparable expression to the HD1 strain was found in isolates 6A, 42MY, 45MY, and 51MY, and much lower expression in isolates Bt-KH58, 85PPb, and 44MY.

Cry protein expression was determined in the five *Bt* isolates (6A, 42MY, 45MY, 51MY, and Bt-KH58) selected on the basis of expressing a Vip3A protein and being toxic to the three insect species tested (Table 2). Expression of Cry proteins was determined by solubilizing the crystals and subjecting them to LC-MS/MS analysis. The results conclusively identified the Cry1Ac protein and a Cry2A-type protein in all isolates, Cry1Ea in 3 of them and Cry1Aa in just one (Table 5). The identity of the Cry2A proteins could not be determined because the Cry proteins within a same subgroup have significant sequence similarity to each other.

**Table 5:** Protein and peptide profiles of *Bt* isolates as obtained by LC-MS/MS.

	Protein				Number of peptides with contribution value > 2	
Isolate	Protein name ^*a*^	Unused ProtScore	Sequence coverage (%)	No. of peptides	Shared peptides^*b*^	Unique peptides^*c*^
**6A**	Cry1Ac	568.03	75.81	611	215	3
	Cry2A	92.95	65.09	71		
**42MY**	Cry1Ac	572.39	64.43	449	232	2
	Cry1Ea	180.36	69.09	372	66	6
	Cry2A	20.86	36.02	24		
**45MY**	Cry1Ac	392.57	78.78	532	120	3
	Cry1Ea	138.46	75.06	343	40	7
	Cry2A	274.67	92.58	352		
**51MY**	Cry1Ac	452.21	79.63	606	161	8
	Cry1Ea	195.91	77.54	440	60	6
	Cry2A	151.04	81.99	171		
**Bt-KH58**	Cry1Aa	594.3	57.57	551	232	2
	Cry1Ac	83.06	60.61	566	29	3
	Cry2A	51.17	42.97	39		

^*a*^ The accession number of the proteins in the database are: Cry1Aa, P0A368; Cry1Ac, P05068; Cry1Ea, Q57458.

^*b*^ Number of peptides with a contribution value higher than 2 that are present in two or more proteins of the Cry1 or Cry2 protein families.

^*c*^ Number of peptides with a contribution value higher than 2 that are present only once in one protein of the Cry family.

### Cloning and sequencing of a new *vip3A* gene

**Fig 3:**
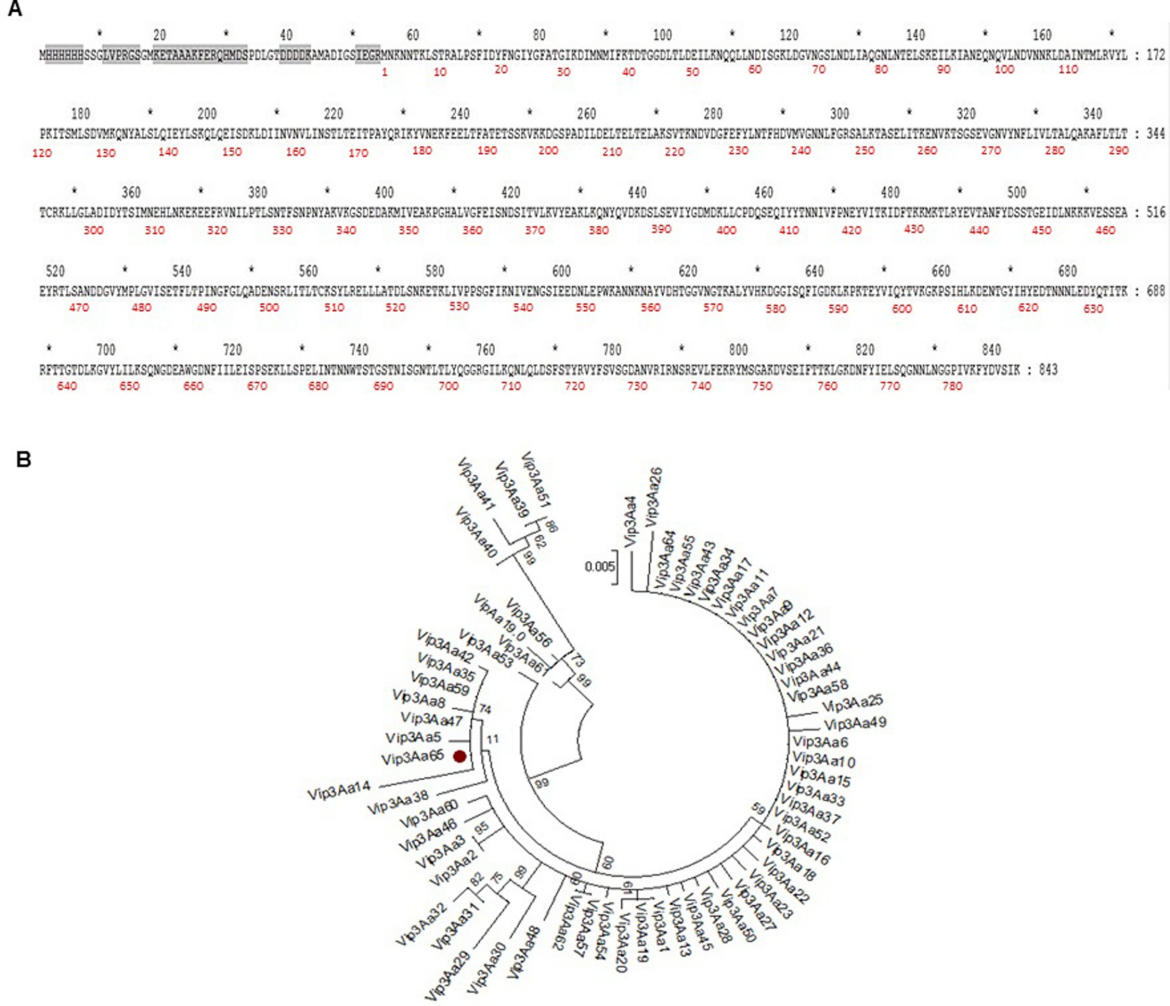
Vip3Aa65 amino acid sequence and phylogenetic tree of Vip3Aa proteins. A) Assembled contig sequence of the Vip3Aa65 expressed protein construct. Gray boxes indicate the sequences for the His-tag, thrombin cleavage site, S-tag, enterokinase cleavage site, and Factor Xa cleavage site. Red numbers below the sequence refer to the amino acid sequence of the Vip3Aa65 protein (from amino acid 1 to 789). B) Maximum likehood tree of the Vip3Aa group performed with the MEGA 6.06 software. Branch lengths represent the number of substitutions per site of the multiple-sequence alignment as a measure of divergence. In addition, the bootstrapping values (number next to the nodes) indicates how many times out of 100% the same branch was observed when repeating the phylogenetic reconstruction on a re-sampled set of your data.

Among the 18 *vip3-*positive isolates, the *Bt* isolate 6A was selected for gene cloning and sequencing because of being one of the isolates expressing more Vip3 protein (Fig 2) and showing high toxicity against *Spodoptera* species in supernatant bioassays (Table 3). The recombinant plasmid DNA was sequenced and the sequence confirmed the presence of the *vip3Aa* gene. The nucleotide sequence, submitted to Genebank data base, was given the accession number MH290720 and the protein was named as Vip3Aa65 by the *Bacillus thuringiensis* Toxin Nomenclature Committee. The amino acid sequence of Vip3Aa65 (Fig 3A) was compared with other Vip3Aa proteins and the degree of similarity is shown in Fig 3B. Vip3Aa65 fell in the cluster of Vip3Aa47, Vip3Aa35 and Vip3Aa59 (which had identical amino acid sequences), though it differed from them by one amino acid difference.

### The Vip3Aa65 protein forms tetramers in solution

**Fig 4:**
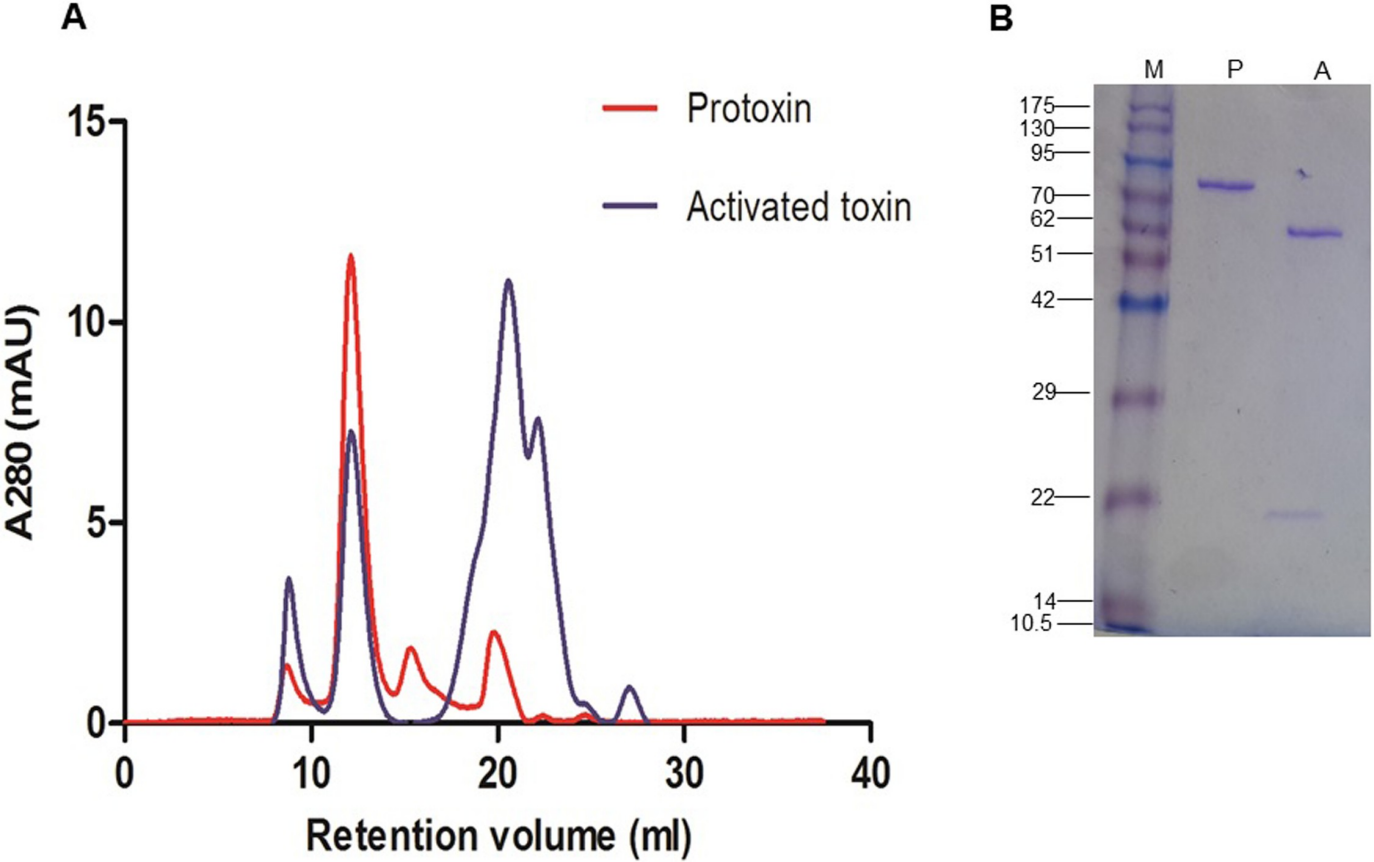
**Oligomeric structure of Vip3Aa65 protoxin and trypsin-activated toxin under native (A) and denaturing (B) conditions**. A) Gel filtration chromatography in a Superdex 200 10/30 GL column. B) SDS-PAGE analysis of the 12 ml peak from the protoxin (P) and the activated toxin (A). M: Molecular weight markers in kDa.

To determine whether the Vip3Aa65 protein adopts an oligomeric structure in solution, the HisTrap purified protein, both as protoxin and as a trypsin-activated toxin, was subjected to gel filtration chromatography. As shown in Fig 4A (red line), after a peak corresponding to aggregates (~9 ml, the void volume of the column), most of the protoxin elutes at 12 ml, corresponding to a globular protein of 346 kDa, which is approx. 4 times the molecular weight of monomeric Vip3Aa65 (88.5 kDa). Just a minor proportion of the protoxin elutes at 15 ml, which corresponds to a globular protein of 80.3 kDa, very close to a monomeric structure of the protein. The peak at 20 ml corresponds to small peptides of around 6 kDa. In the case of the trypsin-activated toxin (Fig 4A, blue line), in addition to small peptides eluting at >20 ml, the main protein peak eluted as a tetramer, with no peak corresponding to the monomeric form.

The protein eluting in the peak at 12 ml, from both the protoxin and the activated toxin, was run on SDS-PAGE (Fig 4B). The denaturing conditions of the SDS-PAGE showed a single band for the protoxin of ~88.5 kD, whereas the activated toxin showed two bands of ~62 kDa and ~19 kDa. This indicates that the Vip3Aa65 protein, either as protoxin or after trypsin activation, is found in a tetrameric form in solution under native conditions and that the oligomer is destroyed under the denaturing conditions of SDS-PAGE. Furthermore, as reported for other Vip3 proteins [24,25,26] the trypsin treatment of Vip3Aa65 gives rise to two main fragments which remain together.

### Characterization of the insecticidal activity of the new Vip3Aa65 protein

The insecticidal activity of Vip3Aa65 was tested against 5 different lepidopteran species and the results are summarized in Table 6. The protein was highly toxic to *G*. *molesta* (LC_50_ = 49 ng/cm^2^) and moderately toxic to the rest of the species (LC_50_ = 496–2660 ng/cm^2^). For comparative purposes, the assays were done side by side with Vip3Aa16, a protein well characterized from the point of view of its insecticidal activity. The two Vip3Aa proteins were similarly active against *H*. *armigera* and *G*. *molesta*, though the Vip3Aa65 protein was found significantly less toxic than the Vip3Aa16 protein against the three species from the genus *Spodoptera*.

**Table 6:** Toxicity of Vip3Aa65 and Vip3Aa16 purified proteins against five lepidopteran species.

Insect species	Protoxin	Slope±SE	LC_50_ (ng/cm^2^)	95% Fiducial limits
***S*. *exigua***	Vip3Aa65	1.24±0.12	496	289–866
	Vip3Aa16	1.02±0.10	79	41–135
***S*. *littoralis***	Vip3Aa65	1.39±0.19	521	307–963
	Vip3Aa16	1.73±0.18	19	11–31
***S*. *frugiperda***	Vip3Aa65	0.52±0.08	2660	1100–10350
	Vip3Aa16	0.53±0.08	168	54–404
***H*. *armigera***	Vip3Aa65	0.78±0.09	1650	890–3670
	Vip3Aa16	0.98±0.12	1330	820–2190
***G*. *molesta***	Vip3Aa65	0.99±0.11	49	22–115
	Vip3Aa16	1.04±0.11	45	26–90

## Discussion

Insect pests belonging to the order of Lepidoptera cause a major damage in crop production [27]. Use of biopesticides is one of the most common methods to control insect pests. *Bt* is a natural insect pathogen and capable of producing Cry and Cyt proteins as well as vegetative insecticidal proteins [2]. Each strain of *Bt* has a varying degree of toxicity against different pests. Therefore, *Bt* collections are a source of new strains with interesting insecticidal properties and also a source for novel genes coding for insecticidal proteins. In the present study, we found that, among 80 *Bt* isolates, 23% of them carried a *vip3* gene. Results from previous studies screening for *vip3* genes show a variable range of incidence, with percentages such as 15% [4], 23% [28], 49% [10], 69.3% [11] and 87% [29] among others. The variable prevalence of *vip3* genes may be due to differences in geographical habitats and environmental conditions. In agreement with other studies showing the tendency of *vip3* genes to be carried out along with *cry1A* and *cry2* genes [10], all our *vip3*-positive isolates had been identified, in previous studies, to carry *cry1* and *cry2* genes [30,31,32,33].

The insecticidal activity of spore/crystal mixtures is an effective way to search for native *Bt* isolates having new entomocidal properties [7,32,34,35,36]. In our study, preliminary assays indicated that some of the 18 selected isolates in the collection had high toxicity against Lepidoptera. It is well known that Cry1 and Cry2 type proteins are toxic to this order of the insects. Hence, the observed toxicity of the tested isolates is presumed to be mainly due to the expression of these type of Cry proteins. When the isolates were further tested in dose-response assays, performed side by side, *S*. *exigua* was found to be more tolerant, in general, than *S*. *littoralis* and *O*. *nubilalis* (Table 2), even to the control strain HD1. Only one isolate (Bt-KH58) exhibited an LC_50_ value that was 3-fold lower than HD1 against *S*. *exigua*. Bt isolates 13MY, 42MY, 45MY and 51MY were highly toxic to *S*. *littoralis*. Likewise, Alper *et al*. [32] found that spore/crystal mixtures of 13MY, 42MY and 45MY isolates showed high activity against another lepidopteran species, *Cadra cautella*. This result strengthens the potential of these isolates to control lepidopteran pests.

The Cry protein composition of parasporal crystals was determined in five selected isolates (6A, 42MY, 45MY, 51MY, Bt-KH58) (Table 2 and Fig 2). LC-MS/MS analysis was able to unambiguously reveal the presence of three Cry1 proteins (Cry1Aa, Cry1Ac, Cry1Ea) and the presence of proteins of the Cry2A family (Table 5). However, due to limitations in this type of analysis and the high homology among Cry proteins, we cannot discard the presence of other proteins in the crystal. A previous study confirms the presence of *cry1Ac* and *cry1Ea* genes, among others, in the 42MY, 45MY and 51MY isolates [32].

The supernatants resulting from culturing the 18 selected *Bt* isolates were tested in bioassays to determine whether they could have insecticidal activity. The results indicated that five isolates were highly toxic (90–97% mortality in the assay conditions), two of them (6A and 45MY) to both *S*. *exigua* and *S*. *littoralis*, and three of them just to *S*. *littoralis* (42MY, 51MY and Bt-KH3) (Table 3). None of them showed high toxicity to *O*. *nubilalis*. The fact that these supernatants lost all activity after being autoclaved, along with the absence of β-exotoxin as revealed by LC-MS/MS, is a strong suggestion of the activity being due to the presence of the Vip3Aa protein. In fact, in agreement with the dot blot assays, isolates 6A, 42MY, 45MY and 51MY showed a high expression of the Vip3Aa protein (Fig 2). The only exception was the Bt-KH3 isolate, which does not express much Vip3Aa protein, though the activity against *S*. *littoralis* was high. Another unexpected result was the moderate activity of some supernatants against *O*. *nubilalis* (up to 56% mortality). Since it is well known that this insect species is non susceptible to Vip3Aa [37,38,39], the mortality observed could be due to another heat-labile component in the supernatant different from Vip3Aa, which would also explain the toxicity of the Bt-KH3 isolate.

PCR sequencing was performed to characterize the *vip3* genes present in our isolates. Our results showed that *vip3Aa* (69%) was the most abundant gene, followed by *vip3Af* (19%) and *vip3Ag* (12%). Similar results were obtained in a previous study [10]. Despite the fact that the results of sequencing the *vip3* genes found in our isolates gave an identity ranging from 94–100% with already reported genes, we decided to clone and characterize the gene from the 6A isolate, in part because its high expression in dot blots, and in part because the high insecticidal activity shown in the supernatant. The cloned and sequenced *vip3Aa65* gene from the 6A isolate had an open reading frame of 2370 bp, encoding a protein of 789 amino acids with a molecular mass of 88.5 kDa. As shown in other Vip3A proteins, Vip3Aa65 occurs mainly in a tetrameric form in solution and activation by trypsin generates two fragments that remain together [24,25,26,40]. Sequence comparison of the Vip3Aa65 protein with other Vip3Aa proteins showed differences among them. The most similar Vip3Aa proteins, which fall in the same cluster, differ from Vip3Aa65 by either one amino acid (Vip3Aa35, Vip3Aa47, Vip3Aa59), two amino acids (Vip3Aa42 and Vip3Aa8), five amino acids (Vip3Aa5) or nine amino acids (Vip3Aa14).

The toxicity of the affinity-purified Vip3Aa65 protein was tested against five important lepidopteran pests. For the sake of comparison, we included Vip3Aa16 as a reference protein which has been used in previous studies [22,23,41,42], which differs from Vip3Aa65 by 9 amino acid residues (Table 7). Although both proteins were similarly toxic to *G*. *molesta* and *H*. *armigera*, significant differences were found in the three tested species of the *Spodoptera* genus (Table 6). The lower insecticidal activity of the Vip3Aa65 protein compared to Vip3Aa16 against *Spodoptera* spp., but not against other insect species, is an indication of the different involvement of some amino acids in the mode of action of the Vip3Aa protein depending on the target insect. Future studies using site-directed mutagenesis of recombinant Vip3Aa65 protein should reveal the contribution of each amino acid to the insecticidal activity of the protein.

**Table 7:** Differences between Vip3Aa65 and Vip3Aa16 amino acid sequences.

	Amino acid positions								
**Protein**	121	358	536	633	755	760	761	776	782
**Vip3Aa16**	L	I	S	N	M	F	E	Y	H
**Vip3Aa65**	I	V	K	T	I	L	G	N	K

Most amino acid differences of these two proteins cluster in the C-terminal part of the protein, from amino acid 755 to 782, with one change adding a negative charge (E to G) and another adding a positive charge (H to K). Other authors have shown that changes at the C-terminal part of the Vip3A proteins may have important effects on the insecticidal activity [43,44,45]. Therefore, it is very likely that these changes in our Vip3Aa65 could be responsible for the difference in activity against the species from the *Spodoptera* genus, though not against other insect species. These differences in susceptibility, among species, to similar Vip3Aa proteins may be due to the occurrence of different types of receptors in the insect species, which may interact with different epitopes in the Vip3Aa protein. Different putative receptor proteins have been proposed for Vip3A proteins, based on ligand blots, depending on the insect studied [46,47,48,49,50].

In conclusion, 18 selected isolates have been characterized for their insecticidal activity of both the parasporal crystal fraction and the culture supernatant, with the result of finding some highly toxic isolates in our collection. Moreover, a new gene (*vip3Aa65*) has been isolated and the activity of the encoded protein was tested against five lepidopteran pests. The combined insecticidal activity of the crystal and supernatant fraction containing the Vip3Aa protein makes these isolates promising candidates for the development of new biopesticides.

## Supporting information

S1 Table Unique peptides for the Cry1Aa, Cry1Ac and Cry1Ea detected by LC-MS/MS analysis. (DOCX)
