# Supplementary material for: Characterization of Bacillus thuringiensis isolates by their insecticidal activity and their production of Cry and Vip3 proteins
Source: PLoS One. 2018 Nov 1;13(11):e0206813. doi: 10.1371/journal.pone.0206813 (PMC6211755; doi:10.1371/journal.pone.0206813)
Supplement: S1 Table — (DOCX) [file pone.0206813.s001.docx]

| **Protein** | **Unique peptides*** | **Isolate** | | | | | |
| --- | --- | --- | --- | --- | --- | --- | --- |
|  |  | **6A** | **42MY** | **45MY** | **51MY** | **Bt-KH58** |  |
| Cry1Ac | EWNPDLDCSCR | X | - | - | - | - |  |
|  | GYIEVPIHFPSTSTR | X | - | X | - | - |  |
|  | (S)AQSPIGK | X | - | - | - | - |  |
|  | LNSSGNNIQNR | - | X | - | X | - |  |
|  | TSSLGNIVGVRN | - | X | - | - | - |  |
|  | (NF)SGTAGVIIDR(F) | - | - | X | X | - |  |
|  | YASVTPIHLNVNWGN(SSIFSN) | - |  | X | X | - |  |
|  | AFTSSLGNIVGVR | - | - | - | X | - |  |
|  | IEVPIHFPSTSTR | - | - | - | X | - |  |
|  | PEVEVLGGER | - | - | - | X | - |  |
|  | PVLENFDGSFR | - | - | - | X | - |  |
|  | VAQLGQGVYR | - | - | - | X | - |  |
|  | GSAQGIER | - | - | - | - | X |  |
|  | IVAQLGQGVYR | - | - | - | - | X |  |
|  | LSHVSMFR | - | - | - | - | X |  |
| Cry1Aa | LSHVTMLSQAAGAVYTLR | - | - | - | - | X |  |
|  | (R)TSPGQISTLR | - | - | - | - | X |  |
| Cry1Ea | EIGESLTSRT | - | X | - | - | - |  |
|  | IAEELPIR(GGE) | - | X | - | - | - |  |
|  | IDTDLIRG | - | X | - | - | - |  |
|  | TIAPSTFPGLNLFYR | - | X | X | - | - |  |
|  | VGPSFENIENSAIR | - | X | X | - | - |  |
|  | VTSHFTGSSQVITTPQYGITANAEPR(R) | - | X | X | - | - |  |
|  | ITVAIGGQIR | - | - | X | X | - |  |
|  | SNSTVATNIALEISR | - | - | X | X | - |  |
|  | TFSYTNFSNPF | - | - | X | X | - |  |
|  | TMEIGESLTSR | - | - | X | - | - |  |
|  | (R)NTIGEFVSLQVNINSPITQR | - | - | - | X | - |  |
|  | (RSEN)ITPTLGINVVQGVGFIQPN(NAEVLYR) | - | - | - | X | - |  |
|  | (RT)IAPSTFPGLNLFYR | - | - | - | X | - |  |
|  |  |  |  |  |  |  |  |

**Table S1.** Unique peptides detected in the *Bt* isolates 6A, 42MY, 51MY and Bt-KH58 by LC/MSMS analysis.

***** The peptide sequences with some amino acids within brackets mean that the peptide has been found with different lengths.
